# Cookies Fortified with *Lonicera japonica* Thunb. Extracts: Impact on Phenolic Acid Content, Antioxidant Activity and Physical Properties

**DOI:** 10.3390/molecules27155033

**Published:** 2022-08-08

**Authors:** Weiwei Cao, Junliang Chen, Linlin Li, Guangyue Ren, Xu Duan, Qian Zhou, Mengli Zhang, Danping Gao, Shanshan Zhang, Xu Liu

**Affiliations:** College of Food and Bioengineering, Henan University of Science and Technology, Luoyang 471023, China

**Keywords:** *Lonicera japonica* Thunb, polyphenols, cookies, antioxidant activity

## Abstract

**Highlights:**

- Cookies with a 4% level of LJ extracts possessed the highest chlorogenic acid content.
- The addition of higher levels of LJ extracts significantly increased higher antioxidant activity of cookies.
- Cookies with a 1% level of LJ extracts had the highest sensory score.

**Abstracts:**

*Lonicera japonica* Thunb. (LJ), as a Caprifoliaceae family plant, is enriched with polyphenols. Cookies supplemented with LJ extracts have the potential to exert antioxidant activity. However, studies on cookies fortified with LJ extracts are scarcely available. Therefore, the effect of LJ extract addition on cookie phenolic acid content, antioxidant activity, color, texture and the sensory score was firstly evaluated. Results suggest that different levels (1–4%) of LJ extracts significantly increased chlorogenic acid content, ranging from 21.96 to 202.65 μg/g. Cookies with a 4% level of LJ extracts possessed the highest activity of scavenging DPPH free radical activity (63.71 μg Vc/g), ABTS free radical activity (415.10 μg Vc/g), and ferric-reducing power of cookies (169.58 μg Vc/g). Further, a decrease in lightness L* and an increase in redness a* were observed in cookies with LJ extract addition. LJ extract addition lowered the hardness of cookies, and 4% level of LJ extracts increased the crispiness of cookies. Cookies with a 1% level of LJ extracts had a higher overall acceptance score (84.33) than that of other levels. Sensory acceptance played a vital role in the selection of the optimal formulation of cookies. Therefore, LJ extracts at 1% level could be an optimal supplement proportion in cookies and increased the antioxidant activity of cookies.

## 1. Introduction

*Lonicera japonica* Thunb. (LJ) belongs to the Caprifoliaceae family, which is widely distributed in Asia including in Japan, China and Korea [1]. LJ has been deemed as a medicinal plant for treating swelling, measles, pneumonia, breast cancer, infection and dysentery [2,3,4]. Moreover, LJ is also used in the food industry such as in healthy tea or beverages. The various medical and food applications of LJ are typically attributed to the bioactive compounds which contains chlorogenic acid and flavonoids [5]. Chlorogenic acid, as the main phenolic acid in LJ, has been reported to possess multiple healthy functions such as antioxidant and anti-inflammatory activities as well as preventing cardiovascular diseases [6]. Herein, LJ extracts have the potential to be widely applied in the healthy food industry to increase food nutrition.

Cookies, as a ready-to-eat bakery product, are popular among consumers of different ages due to good flavor, different varieties, good stability and convenience. Different types of cookies fortified with phenolic acids have attracted more attention due to their health benefits and better quality in recent years [7,8]. For example, cookies with coffee extract residues have higher phenolic acid content, antioxidant activity and lipase inhibitory activity than cookies without coffee extract addition (control cookies), and had similar quality properties to control cookies [9]. Peach pulp fruit incorporation changed the polyphenol profile and improved the antioxidant properties of cookies [10]. The addition of *Tinospora cordifolia* extracts increased the 2,2-diphenyl-1-picrylhydrazyl (DPPH) free radical scavenging activity, the ferric-reducing power (FRAP) and the total polyphenolic content of cookies [11]. Cookies with 10% level of bee pollen had a higher taste score, overall sensory score and antioxidant activity than that for control cookies [12]. Polat et al. reported a 10% and 15% level of germinated lentil extracts increased the hardness and flexibility of crackers [13]. These above studies suggest that the antioxidant activity and quality of cookies can be changed by the addition of phenolic acid extracts. LJ extracts abundant in phenolic acids could exert various health benefits, hence we hypothesized that the incorporation of LJ extracts in cookies could improve the quality and the antioxidant activity of cookies. However, studies on the antioxidant activity and quality of cookies with LJ extract addition are not available to date.

Therefore, the aim of this study was to evaluate the effect of different levels of LJ extracts on the phenolic acid content, antioxidant activity and quality properties including the sensory score, texture and color of cookies.

## 2. Results and Discussion

### 2.1. The TPC of Cookies

**Figure 1:**
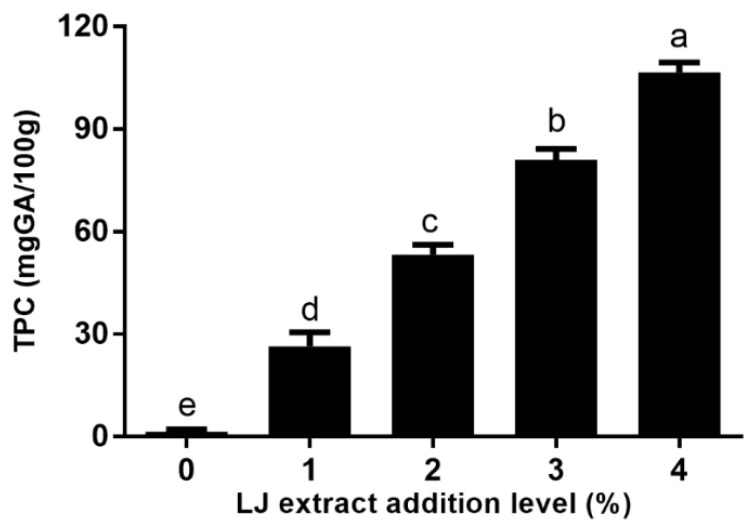
TPC of cookies with LJ extract addition at different levels. Different letters (a–e) represent significant differences, *p* < 0.05.

The effect of different levels of LJ extracts on the TPC of cookies is shown in Figure 1. As shown in Figure 1, the TPC of cookies significantly increased with the increasing levels of LJ extract, which suggests an obvious dose–effect relationship. The TPC of cookies with LJ extract levels of 1%, 2%, 3%, and 4% was 26.44, 53.28, 81.01 and 106.7 mg GA/100 g, respectively. The highest TPC of cookies with a 4% level of LJ extract was 75.11- fold that of control cookies. As LJ extracts are rich in various phenolic acids [14], LJ extracts increased the TPC of cookies, which was in agreement with the results of biscuits fortified with bee pollen rich in polyphenols [15]. In addition, Galvao et al. also reported that cookies with a 5% level of camu-camu coproduct powder have a high TPC of 0.12 mg GA/g, and the TPC of biscuits with *Parinari curatellifolia* peel flour addition was higher than thatfor control cookies [16,17]. Therefore, LJ extracts can be used in cookies and other bakery products to enrich nutritional value of food.

### 2.2. The Antioxidant Activity of Cookies

**Figure 2:**
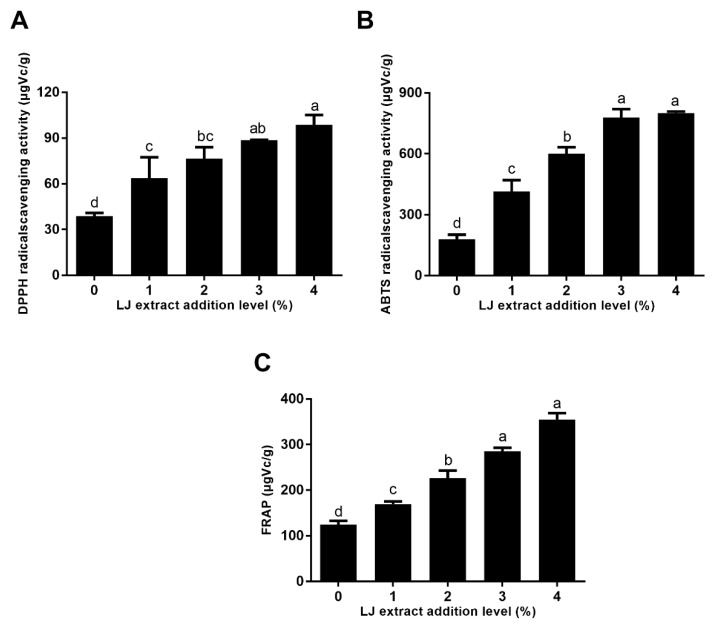
The antioxidant activities for (**A**) DPPH, (**B**) ABTS and (**C**) the FRAP of cookies formulated with LJ extracts at different levels. Different letters (a–d) represent significant differences, *p* < 0.05.

The antioxidant activity of cookies is displayed in Figure 2. As the level of LJ extract added increased from 1 to 3%, the activity of cookies on scavenging DPPH free radical, ABTS free radical and the FRAP was significantly increased, which depended on the antioxidant activity of rich polyphenols in LJ [6]. The trend in the FRAP, the scavenging DPPH and the ABTS free radical activity of cookies with 1–3% levels of LJ extracts was in agreement with the results of the TPC, verifying that the TPC mainly contributed to the antioxidant activity of cookies. However, the three kinds of antioxidant activity of cookies with a 3% level of LJ extracts were not significant compared to that of cookies with a 4% level of LJ extract. The activity of cookies with the lowest LJ extract level (1%) on scavenging DPPH free radical activity, ABTS free radical activity and the FRAP reached 63.71, 415.10, and 169.58 μg Vc/g, respectively. Compared with the control cookies, the DPPH free radical scavenging, ABTS free radical scavenging and the FRAP of cookies with a 4% level of LJ extract increased by 1.55, 3.46 and 1.85-fold, respectively. These results indicated that LJ extract addition remarkably elevated the antioxidant activity of cookies, which further increased the healthy value of novel cookies as functional food. Similar results were also reported in cookies incorporated with *Tinospora cordifolia* (TC) stem powder in terms of the high content of antioxidant compounds [11]. The DPPH and ABTS radical scavenging ability of cookies prepared with pea–wheat flour was relatively higher than biscuits with a whole wheat flour formulation [18]. Therefore, LJ extract addition can further increase the antioxidant activity of cookies and further reduce the oxidative damage caused by free radicals in the body.

### 2.3. Chlorogenic Acid Content in Cookies

**Figure 3:**
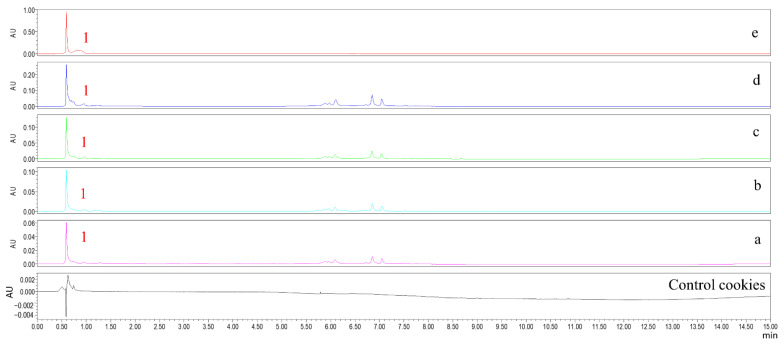
UPLC of chlorogenic acid of cookies. Peak 1 represents chlorogenic acid. a: cookies with a 1% level of LJ extract, b: cookies with a 2% level of LJ extract, c: cookies with a 3% level of LJ extract, d: cookies with a 4% level of LJ extract, and e: chlorogenic acid standard. Control cookies: cookies without LJ extracts.

**Figure 4:**
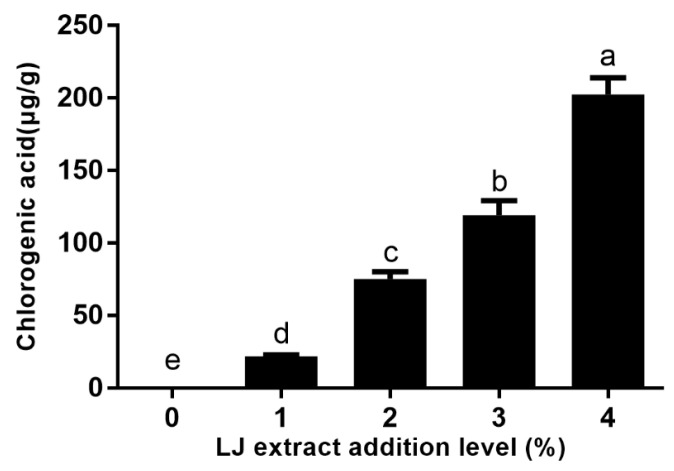
Chlorogenic acid content of cookies with LJ extract addition at different levels. Different letters (a–e) represent significant differences, *p* < 0.05.

The effects of LJ extract addition on chlorogenic acid content in cookies are shown in Figure 3 and Figure 4. The chlorogenic acid content in cookies significantly increased in a dose–effect manner as the level of LJ extracts was increased, and the chlorogenic acid content in control cookies was not detected. The chlorogenic acid content in cookies with 1–4% levels of LJ extracts ranged from 21.96 to 202.65 μg/g. The results provide the explanation for the increase in the TPC and the antioxidant activity of cookies with LJ extracts. Similar results were also found in biscuits enriched with polyphenol extracts from Italian black rice [19]. Compared with cookies with a 1% level of LJ extracts, the chlorogenic acid content in cookies with 2%, 3% and 4% levels of LJ extracts increased by 2.43, 4.43, and 8.23- fold, respectively, which was attributed to LJ extracts being good resources of chlorogenic acid [2]. The order of chlorogenic acid content in cookies with LJ extracts was consistent with the TPC and the antioxidant activity of cookies, which implies that chlorogenic acid plays an important role in the antioxidant activity of cookies with LJ extracts.

### 2.4. Correlation Analysis

**Figure 5:**
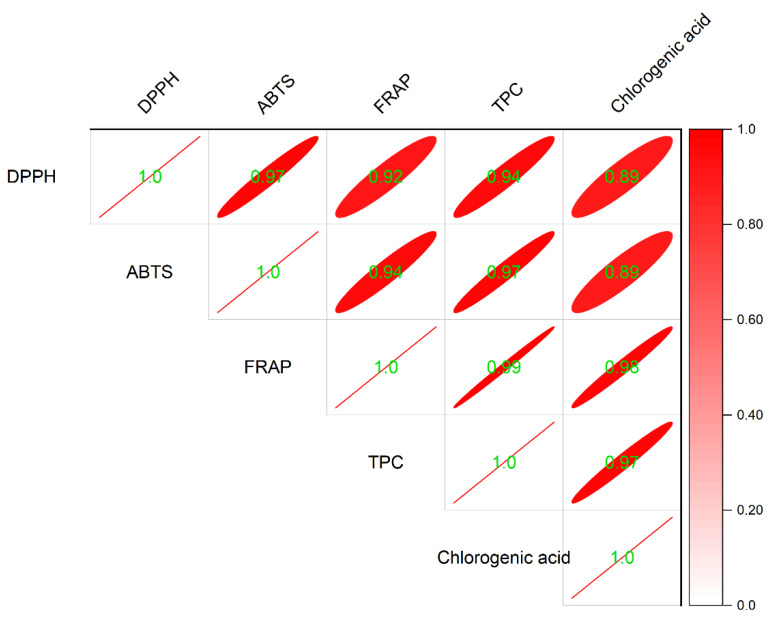
Correlation analysis of the chlorogenic acid content, the TPC and the antioxidant activity of cookies.

The correlation analysis of the chlorogenic acid content, the TPC and the antioxidant activity of cookies are shown in Figure 5. The correlation coefficients between the TPC and the three kinds of antioxidant activity of cookies were all above 0.93 (*p* < 0.05), which suggests that the TPC significantly correlates with the antioxidant activity of cookies with LJ extract addition. Similarly, the correlation between the antioxidant activity and the chlorogenic acid content of cookies was also significant, with correlation coefficients above 0.88. Further, the correlation coefficient between the TPC and the chlorogenic acid content of cookies was 0.97 (*p* < 0.05), further verifying chlorogenic acid as the vital phenolic acid in LJ extracts that contributed to the antioxidant activity of cookies. These results are consistent with the increase in the TPC, the chlorogenic acid content and the antioxidant activity of cookies with different levels of LJ extract addition.

### 2.5. Color of Cookies

Color is a key factor for consumers to evaluate the overall acceptance of cookies. The color analysis of cookies with different levels of LJ extracts is shown in Table 1. The value of lightness L* decreased as the LJ extract level increased, showing that LJ extract addition resulted in a darkening cookie color. The lightness L* value of cookies with LJ extracts ranged from 69.24 to 62.30, all lower than that of control cookies (73.22). This similar phenomenon was consistent with the addition of purple passion fruit epicarp flour and coffee extract residues reducing the brightness of biscuits [7,9]. There was no significant discrepancy among the redness a* (>0) value of cookies with different levels of LJ extracts. However, the redness a* (>0) and yellowness b* (>0) values of cookies with different levels of LJ extracts were both higher than for the control group. The above results were attributed to the color of LJ extract itself being darker than that of wheat flour. Furthermore, LJ polyphenols participated in a Maillard reaction and a caramelization reaction during the baking process. Further, the Tunisian canary palm dates supplement also elevated the yellowness b* (>0) value of biscuits [20]. Therefore, the color change in cookies with LJ extracts should be considered.

### 2.6. Texture Properties of Cookies

As shown in Table 2, the crunch value of cookies with 0–4% levels of LJ extracts ranged from 3.00 to 4.25; however, significant differences were not observed between cookies with different levels of LJ extracts and control cookies. The 1–3% levels of LJ extracts did not affect the crispiness of cookies, but cookies with a 4% level of LJ extracts had the highest value of crispiness (12.00), which made cookies much crispier. The hardness value of cookies decreased as the level of LJ extracts added increased. The hardness value of control cookies was 2061.80 g, while the hardness of cookies with a 4% level of LJ extracts was 1540.32 g. Cookie hardness relies on the gluten network formed by glutenin and gliadin, which also corresponds with the rheological properties of the dough [21]. LJ extracts might change the rheological properties of the dough via the interaction between LJ extracts and proteins containing glutenin and gliadin, which brought about the decrease in cookie hardness. Similarly, the addition of bee pollen decreased the hardness of cookies [15]. Previous studies have presented similar findings, where the hardness of biscuits was reduced with the additional component of quinoa flour [22] and *Parinari curatellifolia* peel flour [19]. Therefore, cookie texture can be altered by LJ extracts.

### 2.7. Sensory Evaluation of Cookies

**Figure 6:**
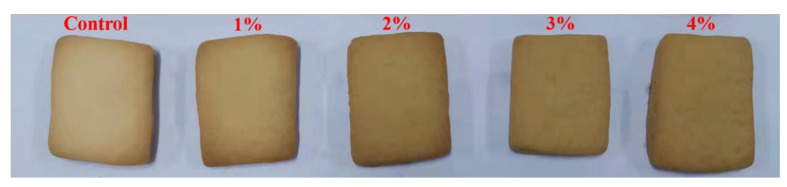
Picture of cookies with LJ extract addition at different levels.

The sensory indicators, including flavor, texture, color, appearance and overall acceptance, are evaluated in Table 3. The overall acceptance score (84.33) of cookies with a 1% level of LJ extracts was equal to that of control cookies (85.67). The scores of flavor, color, appearance and overall acceptance of cookies with a 1% level of LJ extracts was significantly higher than that of cookies with 2–4% levels of LJ extracts. However, there were no significant differences on the texture score of control cookies and cookies with 1–3% levels of LJ extracts, suggesting that an appropriate level of LJ extracts would not destroy the internal structure of cookies. Due to the bitter taste of LJ extracts, cookies with more than a 1% level of LJ extracts brought about an unpleasant flavor. A previous study also suggested that the bitter taste of crackers with olive leaf addition was caused by the presence of oleuropein in olive leaf [23]. The color of cookies with LJ extracts became darker with an increased level of LJ extracts (Figure 6), which caused the lower scores of cookie color. Color, as a key visual factor, is vital for food appearance [24], and the darker color of cookies with a higher proportion of LJ extracts led to lower overall acceptance. When the level of LJ extracts reached 4%, the flavor, color and appearance score of cookies significantly decreased. Cookies with a 4% level of LJ extracts showed the lowest overall acceptance score of 52.67, implying that excess LJ extract addition resulted in the unfavorable quality of cookies. Polat et al. found that crackers enriched with more germinated lentil extracts could lead to unexpected acceptability [13]. Cookies with incorporation of 8% grape pomace had a bitter taste and lower acceptability score, which also supports that the level of phenolic acid extracts should be optimized in cookies [25]. These results suggest that 1% level was the optimal ratio for cookies fortified with LJ extracts.

## 3. Materials and Methods

LJ extracts with 5% chlorogenic acid were purchased from Shaanxi Huike Botanical Development Co., Ltd. (Xi’an, Shaanxi, China). Flour, soda, sugar and butter were ordered from the local supermarket. Chlorogenic acid, Folin–Ciocalteu, 2,4,6-tripyridyl-s-triazine (TPTZ), 2,2′-azinobis-3-ethylbenzothiazoline-6-sulfonic acid (ABTS), and DPPH were ordered from Shanghai yuanye Bio-Technology Co., Ltd. (Shanghai, China). Methanol was ordered from Thermo Fisher Scientific (Waltham, MA, USA). Chlorogenic acid and methanol were of HPLC grade, and other reagents were of analytical grade.

### 3.1. Preparation Cookies with LJ Extracts

The control cookies and cookies with LJ extracts were prepared according to the formulations in Table 4. This formulation was optimized in house and in advance. Firstly, flour, sugar, baking soda and butter were mixed together to prepare the preliminary dough. After mixing for 5 min, LJ extracts (1%, 2%, 3%, and 4%) were supplemented into the dough to form the final dough. For control cookies, LJ extracts were not added into the dough. Lastly, the dough was molded into rectangle shapes with a width of 1.6 cm and a length of 4.0 cm. The above cookies were baked for 20 min at 200 °C in an oven. Cookies were cooled and wrapped in sealed polyethylene packages at 25 °C in a dryer.

### 3.2. Extraction of Phenolic Acids in Cookies

The grinded cookies were mixed with 50% ethanol at 1:7 (*w*/*v*) ratio, and then were extracted in a sonicator for 10 min and centrifuged for 10 min at 8000 rpm/min. The supernatants were collected and stored at −20 °C for total phenolic content (TPC), antioxidant activity and ultra-high-performance liquid chromatography (UPLC) analyses.

### 3.3. Determination of the TPC

The TPC of cookies was measured according to the Folin–Ciocalteu assay with some modifications [9]. The cookie extracts (100 μL) were mixed with 125 μL Folin–Ciocalteu’s reagent, 375 μL sodium carbonate solution and 1500 μL distilled water. The mixture was placed in the dark for 30 min and the absorbance at 750 nm was measured using a UV spectrophotometer. Gallic acid (GA) equivalent was used to calculate the TPC of cookies.

### 3.4. Determination of DPPH Free Radical Scavenging Activity

The DPPH free radical scavenging activity of cookies was determined according to the method reported by Sferrazzo et al. [26] with some modifications. DPPH solution (900 μL) was added to 100 μL cookie extracts and the mixture reacted for 10 min in the dark. The absorbance was determined at 517 nm with a UV spectrophotometer. The DPPH free radical scavenging activity was expressed using the Vc equivalent.

### 3.5. Determination of ABTS Free Radical Scavenging Activity

The ABTS free radical scavenging activity of cookies was analyzed using the method described by Dunford et al. [27]. The ABTS reaction solution (800 μL) reacted with 200 μL cookie extracts for 20 min in the dark. The absorbance was determined at 734 nm with a UV spectrophotometer, and the Vc equivalent was chosen to express the ABTS free radical scavenging activity.

### 3.6. Determination of the FRAP

The FRAP of cookies was performed using the method described by Figueroa et al. reported [28]. The FRAP reaction solution (800 μL) was mixed with 200 μL cookie extracts, and the mixture reacted for 20 min in the dark. The absorbance at 593 nm was measured using a spectrophotometer and the FRAP of cookies was calculated using the Vc equivalent.

### 3.7. UPLC Analysis

The solution of cookie extracts in Section 2.2 was filtered through a 0.22 μm filter membrane and further used for UPLC analysis. The Waters UPLC H-Class system equipped with a diode array detector was employed to determine chlorogenic acid content. Chromatographic separation was performed on a Waters BEH C18 column (50 mm × 2.1 mm, 1.7 μm) at 25 °C. The mobile phase was a mixture of ultrapure water (phase A) and methanol (phase B), flowing at 0.2 mL/min in gradient conditions as follows: 0–3 min, 10% B; 3–6 min, 80% B; 6–10 min, 80% B; 10–13 min, 10% B; 13–15 min, 10% B. The injection volume was 2 µL. The chromatogram spectra were recorded at 325 nm for quantification of chlorogenic acid in cookies. The chlorogenic acid standard solution (100, 200, 300, 400, and 600 μmol/L) was used to obtain the standard curve of chlorogenic acid to calculate the content of chlorogenic acid in cookies.

### 3.8. Color of Cookies

The cookie color parameters of L*, a*, and b* were measured using Xrite color i5 (X-Rite, Grand Rapids, MI, USA). L* represents lightness, +a* represents redness and −a* represents greenness. +b* represents yellowness and −b* represents blueness.

### 3.9. Textural Properties of Cookies

The texture indicators of crunch, crispy and hardness were measured using a TA.XT Express texture analyzer (Stable Micro Systems, Godalming, UK). The parameters of the texture analyzer were as follows: probe type of p/2, pretest speed of 1.0 mm/s, test speed of 1.0 mm/s, post-speed of 10.0 mm/s, strain distance of 3.5 mm, and trigger force of 20.0 g.

### 3.10. Sensory Analysis of Cookies

Cookies were evaluated by 10 assessors (five males and five females with ages ranging from 19 to 40) under white light in individual rooms after these assessors were trained to distinguish the variations in sensory attributes among different cookies to form a standardize assessment. Cookies coded with a random number in a plastic container were served to assessors. To avoid errors, all the assessors were required to rinse their palate with water between evaluating cookies with different formulations. A hedonic scale of one hundred points was adopted to evaluate overall acceptance including flavor (30 scores), texture (30 scores), color (20 scores), and appearance (20 scores).

### 3.11. Statistical Analysis

Statistical significance was determined by one-way ANOVA followed by using SPSS software (version 16.0, SPSS Inc., Chicago, IL, USA). The correlation analysis results of the chlorogenic acid content, the TPC and the antioxidant activity of cookies were expressed as Pearson correlation coefficients by SPSS software (version 16.0, SPSS Inc., Chicago, IL, USA). Results are expressed as the means ± SD, and all the measurements were carried out in triplicate. *p* < 0.05 was considered significant.

## 4. Conclusions

This study firstly developed cookies with LJ extracts and assessed the characteristics of cookie as influenced by 1–4% levels of LJ extracts. Due to the high chlorogenic acid content in LJ extracts, the phenolic acid content and the antioxidant activity of cookies with LJ extract fortification increased. Unfortunately, only chlorogenic acid was identified as the main phenolic acid in cookies with LJ extracts, due to the unavailability of more polyphenol standards and a limited research budget. Further studies on other polyphenols in cookies with LJ extracts should be carried out in the future to reveal its health function. LJ extracts significantly decreased the color parameter L * and the hardness of cookies, which contributed to the overall acceptance score change in cookies. Cookies with a 1% level of LJ extracts exhibited the highest score for flavor, color and overall acceptance. It is also worth noting that cookies with a 4% level of LJ extracts had the highest antioxidant capacity with the lowest sensory score. This was because the sensory score of cookies depended on the flavor, color, texture, and appearance affected by LJ extract addition. Therefore, the best formulation of cookies should be selected based on the sensory score regardless of antioxidant activity. Thus, this study proved that LJ extract is an innovative ingredient that can be used in functional cookies.

## Figures and Tables

**Table 1:** Color parameters of cookies.

LJ Extract Level	L* (Lightness)	a* (Redness)	b* (Yellowness)
0%	73.22 ± 1.13 ^d^	4.60 ± 0.32 ^a^	30.91 ± 0.91 ^a^
1%	69.24 ± 0.39 ^c^	5.49 ± 0.21 ^b^	35.19 ± 1.10 ^b^
2%	64.46 ± 1.21 ^b^	5.50 ± 0.13 ^b^	36.81 ± 0.42 ^c^
3%	64.24 ± 0.64 ^b^	5.63 ± 0.12 ^b^	36.87 ± 0.09 ^c^
4%	62.30 ± 0.26 ^a^	5.68 ± 0.12 ^b^	37.48 ± 0.22 ^c^

L* represents lightness, a* (>0) represents redness and b* (>0) represents yellowness. Different superscript lowercase letters (a–d) in the same column represent significant differences, *p* < 0.05.

**Table 2:** Textural properties of cookies.

LJ Extract Level	Crunch	Crispy	Hardness
0	3.00 ± 1.52 ^a^	8.75 ± 0.96 ^a^	2061.80 ± 196.77 ^c^
1%	3.50 ± 1.92 ^a^	10.00 ± 1.41 ^ab^	1995.75 ± 129.81 ^b^
2%	3.75 ± 1.26 ^a^	10.25 ± 1.50 ^ab^	1968.08 ± 138.69 ^ab^
3%	3.75 ± 1.50 ^a^	11.00 ± 2.45 ^ab^	1786.67 ± 58.16 ^a^
4%	4.25 ± 0.50 ^a^	12.00 ± 1.63 ^b^	1540.32 ± 72.06 ^a^

Different superscript lowercase letters (a–c) in the same column represent significant differences, *p* < 0.05.

**Table 3:** Total sensory score analysis of cookies.

LJ Extract Level	Flavor	Texture	Color	Appearance	Overall Acceptance
0%	27.67 ± 0.58 ^a^	22.67 ± 2.08 ^ab^	15.67 ± 0.58 ^a^	18.33 ± 0.58 ^a^	84.33 ± 1.53 ^a^
1%	27.67 ± 0.58 ^a^	26.00 ± 1.00 ^a^	15.67 ± 1.53 ^a^	16.33 ± 1.15 ^ab^	85.67 ± 3.21 ^a^
2%	21.67 ± 2.89 ^b^	25.00 ± 1.00 ^a^	13.33 ± 0.58 ^b^	15.33 ± 0.58 ^b^	75.33 ± 3.21 ^b^
3%	20.33 ± 1.53 ^b^	23.33 ± 2.89 ^ab^	11.67 ± 0.58 ^b^	15.67 ± 2.31 ^b^	71.00 ± 4.36 ^b^
4%	11.67 ± 3.79 ^c^	20.33 ± 2.52 ^b^	9.33 ± 1.15 ^c^	11.33 ± 1.53 ^c^	52.67 ± 3.79 ^c^

Different superscript lowercase letters (a–c) in the same column represent significant differences, *p* < 0.05.

**Table 4:** The raw material formulation of cookies.

Ingredients	Formulations				
	Control	1%	2%	3%	4%
Wheat flour	100	99	98	97	96
LJ extracts	0	1	2	3	4
Sugar	25	25	25	25	25
Butter	30	30	30	30	30
Baking soda	0.2	0.2	0.2	0.2	0.2

## Data Availability

The datasets generated for this study are available on request from the corresponding author.

## Sample Availability

Samples of the compounds are available from the authors upon request.
